# *Streptococcus pyogenes emm* Type 3.93 Emergence, the Netherlands and England

**DOI:** 10.3201/eid3102.240880

**Published:** 2025-02

**Authors:** Matthew A. Davies, Brechje de Gier, Rebecca L. Guy, Juliana Coelho, Alje P. van Dam, Robin van Houdt, Sébastien Matamoros, Marit van den Berg, Patrick E. Habermehl, Kartyk Moganeradj, Yan Ryan, Steve Platt, Henry Hearn, Eleanor Blakey, Darren Chooneea, Bart J.M. Vlaminckx, Theresa Lamagni, Nina M. van Sorge

**Affiliations:** Amsterdam University Medical Center, Amsterdam, the Netherlands (M.A. Davies, A.P. van Dam, R. van Houdt, S. Matamoros, M. van den Berg, P.E. Habermehl, N.M. van Sorge); National Institute for Public Health and the Environment, Bilthoven, the Netherlands (B. de Gier); UK Health Security Agency, London, UK (R.L. Guy, J. Coelho, K. Moganeradj, Y. Ryan, S. Platt, H. Hearn, E. Blakey, D. Chooneea, T. Lamagni); University Medical Center Utrecht, Utrecht, the Netherlands (B.J.M. Vlaminckx)

**Keywords:** *Streptococcus pyogenes*, streptococci, pneumonia, meningitis/encephalitis, bacteria, epidemiology, invasive infection, genomic inversion, Netherlands, England

## Abstract

A global increase in the incidence of invasive group A *Streptococcus* (iGAS) infections was observed after lifting of COVID-19 related restrictions in 2022, and type M1_UK_ dominated in many countries. After seasonal declines in iGAS incidence during the summer of 2023, simultaneous, rapid expansion of a previously rare *emm* type 3.93 was seen beginning in November, increasing to 20% of all cases in England and 60% of all cases in the Netherlands within 4 months. *emm*3.93 was associated with iGAS in children 6–17 years of age and with increased risk for pneumonia or pleural empyema and meningitis in both countries. No excess risk of death was identified for *emm*3.93 compared with other types. Genomic analysis of historic and contemporary *emm*3.93 isolates revealed the emergence of 3 new clades with a potentially advantageous genomic configuration. Our findings demonstrate the value of molecular surveillance, including long-read sequencing, in identifying clinical and public health threats.

In several countries, including the Netherlands and England, an increase in invasive group A *Streptococcus* (iGAS) infections was observed after lifting of COVID-19 restrictions during 2022 and 2023 (*1*,*2*). The reason for this sudden upsurge has yet to be completely explained but likely resulted from a perfect storm scenario: an increased population susceptibility to *Streptococcus pyogenes* infections after 2 years of reduced circulation that coincided with an increased incidence of predisposing viral infections and further expansion of the *emm*1.0 sublineage M1_UK_ (*3*), which produces increased amounts of the virulence factor and streptococcal pyrogenic exotoxin A compared with its progenitor, M1_global_ (*3*). Similar to the case in other countries, *emm*1.0 was the dominant *emm* type in the Netherlands and England (*1*,*4*,*5*). However, beginning in June 2023, the proportion of *emm*1.0 among clinical *S. pyogenes* isolates diminished in the Netherlands and England, coinciding with a seasonal decrease in iGAS incidence. An increase in a previously rare type, *emm*3.93, was identified among iGAS patients in the Netherlands and England beginning in November 2023. We report a cross-border increase in iGAS with increased infections in particular age groups and specific clinical manifestations related to an *emm*3.93 variant that displays a previously unreported genome configuration.

## Methods

### *S. pyogenes* Isolate Collection, *emm* Typing, and Genome Sequencing

Beginning in April 2022, all medical microbiology laboratories in the Netherlands were asked to submit *S. pyogenes* isolates from patients with iGAS disease (defined as either the detection of *S. pyogenes* from a normally sterile site or from a nonsterile site in combination with a clinical manifestation of iGAS) to the Netherlands Reference Laboratory for Bacterial Meningitis (NRLBM; Amsterdam, the Netherlands) for *emm* typing as part of national bacterial surveillance. Isolates are submitted with limited patient information (patient birth year, sex, and postal code). In addition to recent isolates from prospective bacterial surveillance, we screened an existing bacterial isolate collection (*4*) consisting of *S. pyogenes* carriage isolates (2009–2023, n = 349) and iGAS disease (2009–2019, n = 272) for the presence of *emm*3.93. In England, medical microbiology laboratories were asked to submit *S. pyogenes* isolates to the UK Health Security Agency (London, UK) reference laboratory for *emm* typing as part of active surveillance.

We conducted *emm* genotyping according to US Centers for Disease Control and Prevention protocol (*6*) by using conventional PCR amplification and Sanger sequencing of a 180-bp domain encoding the 50 hypervariable codons that determine *emm* subtype. The *emm*3.93 sequence and information were provided by the National Health Service (NHS) of Greater Glasgow and Clyde, Scotland, UK. We found or generated short-read sequence (Illumina) data for 104 *emm*3.93 isolates, including 11 strains from the Netherlands, 9 from New Zealand, and 84 from England. In addition, we conducted long-read (Oxford Nanopore) sequencing on 14 of the *emm*3.93 isolates, 5 from the Netherlands and 9 from England, for hybrid assembly of the complete genome.

Raw sequencing data and complete genomes are available from the National Center for Biotechnology Information BioProject database (accession nos. PRJNA1125189, PRJEB43915, PRJEB13551, and PRJNA1100230). Sample accession numbers are provided (Appendix Table 1).

### Genome Assembly

We processed raw Illumina reads by using Trimmomatic v0.39 (*7*) to remove adaptor sequences and bases of insufficient quality with the parameters ILLUMINACLIP:TruSeq3-SE:2:30:10 LEADING:3 TRAILING:3 SLIDINGWINDOW:4:15 MINLEN:36. We de novo assembled trimmed reads into scaffolds by using SPAdes v3.15.5 (*8*) and assessed assembly quality by using QUAST v5.0.2 (*9*). We processed raw Nanopore reads by using Porechop v0.2.4 (*10*) with default parameters and assembled the reads by using Flye v2.9.2 (*11*) with parameters -g 5.6m and -i 2. We created closed genomes by using hybrid assembly via Unicycler v0.4.8 (*12*) with default parameters.

### Detection of Genomic Rearrangements

To determine the presence of the genomic inversion, we annotated complete genomes by using bakta (*13*) then aligned and visualized the genomes by using EasyFig (*14*) and its default use of blastn (*15*). We determined the location of prophage sequences MGAS315.1 (RefSeq accession no. NC_004584.1) and MGAS315.2 (RefSeq accession no. NC_004585.1) by using blastN with default parameters. We produced whole genome alignment dotplots by using the web interface version of D-GENIES (*16*) via the built-in aligner, Mashmap v2.0.

### Core Single-Nucleotide Polymorphism Phylogenetic Analysis

We created a core single-nucleotide polymorphism (SNP) alignment by calling SNPs by using snippy v4.6.0 against the reference genome MGAS315 (RefSeq accession no. NC_004070.1) with default parameters. We used Gubbins v2.3.4 (*17*) to detect and mask sites of recombination and performed a phylogenetic inference by maximum likelihood on the subsequent alignment by IQ-TREE (*18*) using the general time-reversible plus gamma plus invariate sites substitution model and visualizing in iTOL v6.9 (*19*). We determined the lineage-defining SNPs to be synonymous, nonsynonymous, or insertion or deletion by using snippy. We created the SNP distance matrix by using SNP-dists v0.8.2 and performed the hierarchical clustering and visualization by using the R package pheatmap (The R Project for Statistical Computing).

### Clinical Information of iGAS Patients

In the Netherlands, clinical manifestations of iGAS with *S. pyogenes* cultured from any site are notifiable by law since January 2023. Pseudonymized notifications, which contain information on the clinical manifestations, onset, and *emm* type (when available), are analyzed by the National Institute for Public Health and the Environment (Bilthoven, the Netherlands). Unknown *emm* types are supplemented by probabilistic linkage of notifications to the NRLBM data on the basis of birth year, gender, postal code, and date of diagnosis.

In England, iGAS has been a notifiable disease since April 2010 (*20*). Notifications include date of birth, sex, postal code, and date of specimen collection. Clinical manifestation information is not collected, but the focus of infection is inferred from the specimen type, in particular, cerebrospinal fluid, pleural fluid, and synovial fluid specimens. All iGAS notifications were submitted to the NHS Demographic Batch Tracing Service to identify the date of death (*21*). We calculated all-cause death by using all deaths within 7 days of a positive iGAS specimen, including all postmortem diagnoses. We used multivariable logistic regression to assess all-cause death and to compare *emm*3.93 with other contemporaneous *emm* types. We retrieved antimicrobial susceptibility testing results from routine laboratory surveillance data.

### Ethics Statement

This study was conducted in accordance with the European Statements for Good Clinical Practice and the Declaration of Helsinki of the World Medical Association. In the Netherlands, invasive *S. pyogenes* isolates were collected as part of routine care. This study was not reviewed by an ethics review board because it is based on anonymized surveillance data. UK Health Security Agency have legal permission under Regulation 3 of the Health Service (Control of Patient Information) Regulations 2002 to process patient identifiable information without consent. This process considers the ethics and purpose of collecting and analyzing the data; therefore, ethics approval was not separately sought for this work.

## Results

### *emm*3.93 Epidemiology Among iGAS Patients

**Figure 1:**
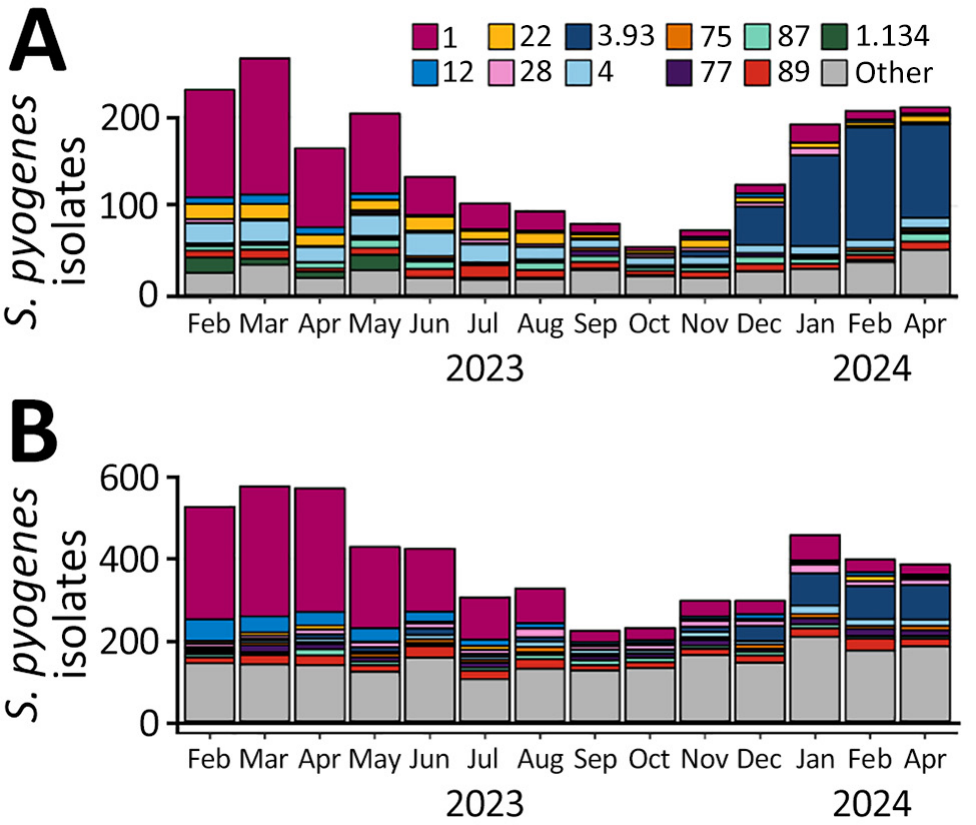
Absolute number of *Streptococcus pyogenes* isolates recovered from patients with invasive group A *Streptococcus*, by *emm* type and month, February 1, 2023–March 31, 2024. A) The Netherlands; B) England.

**Figure 2:**
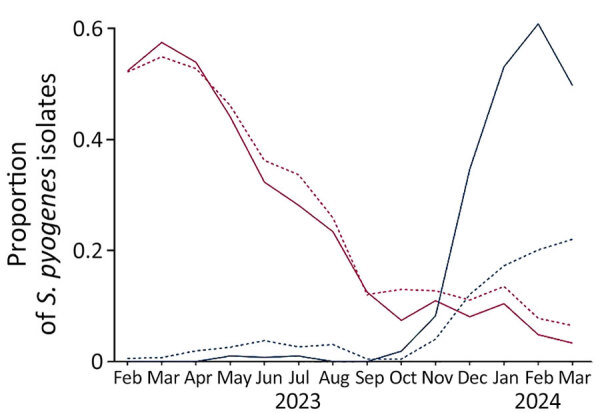
Proportion of *emm* types 1.0 and 3.93 among all typed invasive *Streptococcus pyogenes* isolates in the Netherlands and England, February 1, 2023–March 31, 2024.

We extracted iGAS notifications with disease onset during November 1, 2023–March 31, 2024, from the notification databases of the Netherlands on April 11, 2024 (798) cases and England on April 12, 2024 (1,510 cases). Of reported cases, 665 (83.3%) isolates from the Netherlands and 1,351 (89.5%) isolates from England had a known *emm* type. In both countries, the monthly number of submitted *S. pyogenes* isolates from iGAS patients showed a seasonal decrease during June–October 2023, coinciding with a decrease in the proportion of *emm*1.0 isolates (Figure 1). Beginning in November 2023, iGAS cases increased in line with usual seasonal patterns (Figure 1). Of note, the proportion of *emm*3.93 among typed iGAS isolates in the Netherlands increased from 8% (6/73) in November 2023 to 61% (126/207) in February 2024 and in England from 4% (8/219) in November 2023 to 24% (66/274) in March 2024 (Figure 1, 2). From available *S. pyogenes* isolates from the Netherlands in iGAS patients and asymptomatic carriers (2009–2019), *emm*3.93 was rare, with only 6/577 (1%) typed strains. Similarly, *emm*3.93 only constituted 3% (400/11,194) of *S. pyogenes* isolates in England during 2016–2019. A short upsurge in prevalence to 9% (224/2,597) was seen in 2018, but afterward, *emm*3.93 strains decreased to <1% of typed isolates per year (*22*). Phenotypic antimicrobial susceptibility testing demonstrated low resistance to clindamycin (1.4%, 95% CI 0.0%–7.4%), erythromycin (1.4%, 95% CI 0.0%–7.5%), or tetracycline (0%, 95% CI 0%–4.4%) in *emm*3.93 specimens in England during November 2023–March 2024.

### Clinical Manifestations and Case-Fatality Rate of *emm*3.93 Compared with Other *emm* Types

A multivariable logistic regression model with covariates age group, sex, and month of disease onset revealed that children 6–17 years of age had higher risk for iGAS caused by *emm*3.93 than for other *emm* types in both countries (Table 1). In addition, in England, *emm*3.93 also affected children 0–5 years of age more often (adjusted odds ratio [aOR] 1.7, 95% CI 1.1–2.8) (Table 1). Type *emm*3.93 was recovered less often from patients 18–44 years of age in both countries (Table 1). There was a slight association of *emm*3.93 with female sex compared with other types (aOR 1.4, 95% CI 1.0–1.8) in England, which was not observed in the Netherlands (Table 1).

**Table 1:** Characteristics of patients with invasive group A *Streptococcus* infection in the Netherlands and England caused by *emm*3.93 or other *emm* types during November 1, 2023–March 31, 2024*

**Characteristic**	The Netherlands					England				
*emm*3.93, no. (%)	Other *emm* type, no. (%)	aOR (95% CI)	p value	*emm*3.93, no. (%)	Other *emm* type, no. (%)	aOR (95% CI)	p value
No. patients	319	346				240	1,158		
Age group, y									
0–5	23 (7.2)	17 (4.9)	1.5 (0.7–3.2)	0.2675		30 (12.5)	93 (8.0)	1.7 (1.1–2.8)	0.030
6–17	33 (10.3)	16 (4.6)	2.4 (1.2–5.0)	0.0139		30 (12.5)	60 (5.2)	2.4 (1.4–3.9)	0.001
18–44	65 (20.4)	112 (32.4)	0.6 (0.4–0.9)	0.0204		31 (12.9)	284 (24.5)	0.6 (0.4–0.9)	0.022
45–64	76 (23.8)	84 (24.3)	0.9 (0.6–1.3)	0.5046		63 (26.3)	258 (22.3)	1.4 (0.9–2.0)	0.103
>65	122 (38.2)	117 (33.8)	Referent			86 (35.8)	463 (40.0)	Referent	
Sex									
M	151 (47.3)	159 (46.0)	Referent			123 (51.3)	667 (57.6)	Referent	
F	168 (52.7)	187 (54.0)	1.1 (0.8–1.5)	0.5416		115 (47.9)	477 (41.2)	1.4 (1.0–1.8)	0.033
STSS									
No	265 (88.9)	318 (95.2)	Referent						
Yes	33 (11.1)	16 (4.8)	2.2 (1.1–4.3)	0.0225					
Necrotizing fasciitis									
No	279 (93.6)	302 (90.4)	Referent						
Yes	19 (6.4)	32 (9.6)	0.7 (0.4–1.4)	0.3468					
Puerperal fever or sepsis									
No	270 (90.6)	273 (81.7)	Referent						
Yes	28 (9.4)	61 (18.3)	0.5 (0.3–0.8)	0.004					
Meningitis†									
No	286 (96.0)	328 (98.2)	Referent			228 (98.3)	1,058 (99.9)	Referent	
Yes	12 (4.0)	6 (1.8)	2.7 (0.9–8.8)	0.0818		4 (1.7)	1 (0.1)	28.6 (2.6–320.1)	0.006
Bone or joint infection‡									
No	271 (90.9)	312 (93.4)	Referent			221 (95.3)	1,006 (95.0)	Referent	
Yes	27 (9.1)	22 (6.6)	1.2 (0.6–2.4)	0.586		11 (4.7)	53 (5.0)	1.1 (0.6–2.3)	0.858
Pneumonia/pleural empyema§									
No	217 (72.8)	298 (89.2)	Referent			218 (94.3)	1,039 (98.1)	Referent	
Yes	81 (27.2)	36 (10.8)	2.9 (1.9–4.6)	<0.001		14 (6.0)	20 (1.9)	3.4 (1.6–73)	0.001
Cardiovascular infection									
No	297 (99.7)	329 (98.5)	Referent						
Yes	1 (0.3)	5 (1.5)	0.2 (0.0–1.8)	0.2252					
Skin and soft tissue infection									
No	260 (87.2)	264 (79.0)	Referent						
Yes	38 (12.8)	70 (21.0)	0.5 (0.3–0.8)	0.0032					
Sepsis									
No	231 (77.5)	240 (71.9)	Referent						
Yes	67 (22.5)	94 (28.1)	0.7 (0.5–1.0)	0.055					
Month									
2023 Nov	4 (1.3)	63 (18.2)	Referent			9 (3.8)	218 (18.8)	Referent	
2023 Dec	60 (18.8)	74 (21.4)	12.5 (4.8–43.2)	<0.0001		44 (18.3)	223 (19.3)	4.7 (2.2–9.9)	<0.0001
2024 Jan	86 (27.0)	73 (21.1)	19.1 (7.3–65.6)	<0.0001		61 (25.4)	258 (22.3)	5.7 (2.8–11.8)	<0.0001
2024 Feb	102 (32.0)	69 (19.9)	24.1 (9.3–82.6)	<0.0001		62 (25.8)	227 (19.6)	6.3 (3.1–13.2)	<0.0001
2024 Mar	67 (21.0)	67 (19.4)	14.8 (5.7–51.2)	<0.0001		64 (26.7)	232 (20.0)	6.7 (3.2–13.8)	<0.0001

*Odds ratios for age group, sex, and month are derived from a multivariable model containing those 3 variables. Odds ratios for clinical manifestations are adjusted for age group, sex, and month, but the puerperal fever or sepsis aOR is adjusted for month only. aOR, adjusted odds ratio; STSS, streptococcal toxic shock syndrome. †Cerebral spinal fluid specimens used as proxy in data from England. ‡Joint or synovial fluid specimens used for data from England. §Pleural specimens used as proxy for data from England.

Regarding clinical manifestations, *emm*3.93 was associated with pneumonia or pleural empyema in both countries and with streptococcal toxic shock syndrome (aOR 2.2, 95% CI 1.1–4.3) in the Netherlands (Table 1). Furthermore, *emm*3.93 was associated with an increased risk for meningitis (Table 1), which was significant in England (aOR 28.6, 95% CI 2.6–320.1) but not in the Netherlands (aOR 2.7, 95% CI 0.9–8.8). In the Netherlands, *emm*3.93 was less often recovered from patients with skin or soft tissue infections or puerperal iGAS infection (aOR 0.5, 95% CI 0.3–0.8) (Table 1).

All-cause 7-day case-fatality rate data were available for England only. Case-fatality rate for *emm*3.93 was 13.8% (95% CI 9.6%–18.9%, 32/232) compared with 12.0% (95% CI 10.2%–14.1%, 127/1,059) for other types during November 2023–March 2024. A multivariable logistic regression adjusting for age, sex, specimen month, and *emm* type (*emm*3.93 or other) did not identify a significant difference for 7-day deaths between types (aOR 1.2, 95% CI 0.8–1.8); however, significant differences were noted by age, group, and sex (Table 2).

**Table 2:** Characteristics of patients with invasive group A *Streptococcus* infection caused by *emm*3.93 or other *emm* types according to whether all-cause death was recorded within 7 days of diagnosis, November 1, 2023–March 31, 2024, England*

Characteristics	Died within 7 days, no. (%)	Alive at 7 days, no. (%)	aOR (95% Cl)	p value
No. patients	159	1,132		
*emm* type				
Other	127 (12.0)	932 (88.0)	Referent	
*emm*3.93	32 (13.8)	200 (86.2)	1.2 (0.8–1.8)	0.501
Age group, y				
0–17	11 (5.7)	182 (94.3)	0.3 (0.1–0.5)	<0.0001
18–44	10 (3.5)	276 (96.5)	0.2 (0.1–0.3)	<0.0001
45–64	42 (14.4)	250 (85.6)	0.7 (0.5–1.1)	0.146
>65+	96 (18.5)	424 (82.5)	Referent	
Sex				
M	77 (10.6)	651 (89.4)	Referent	
F	82 (14.6)	481 (85.4)	1.4 (1.0–2.0)	0.045
Month				
2023 Nov	24 (11.4)	187 (88.6)	Referent	
2023 Dec	32 (12.8)	218 (87.2)	1.1 (0.6–2.0)	0.663
2024 Jan	44 (14.9)	251 (85.1)	1.3 (0.8–2.3)	0.339
2024 Feb	34 (12.6)	237 (87.4)	1.2 (0.7–2.1)	0.583
2024 Mar	25 (9.5)	239 (90.5)	0.8 (0.4–1.5)	0.503

*Odds ratios for *emm* type, age group, se,x and month are derived from a multivariable model containing these 4 variables. aOR, adjusted odds ratio.

### Genomic Analysis of *emm*3.93 Isolates in 3 Emergent Clades

**Figure 3:**
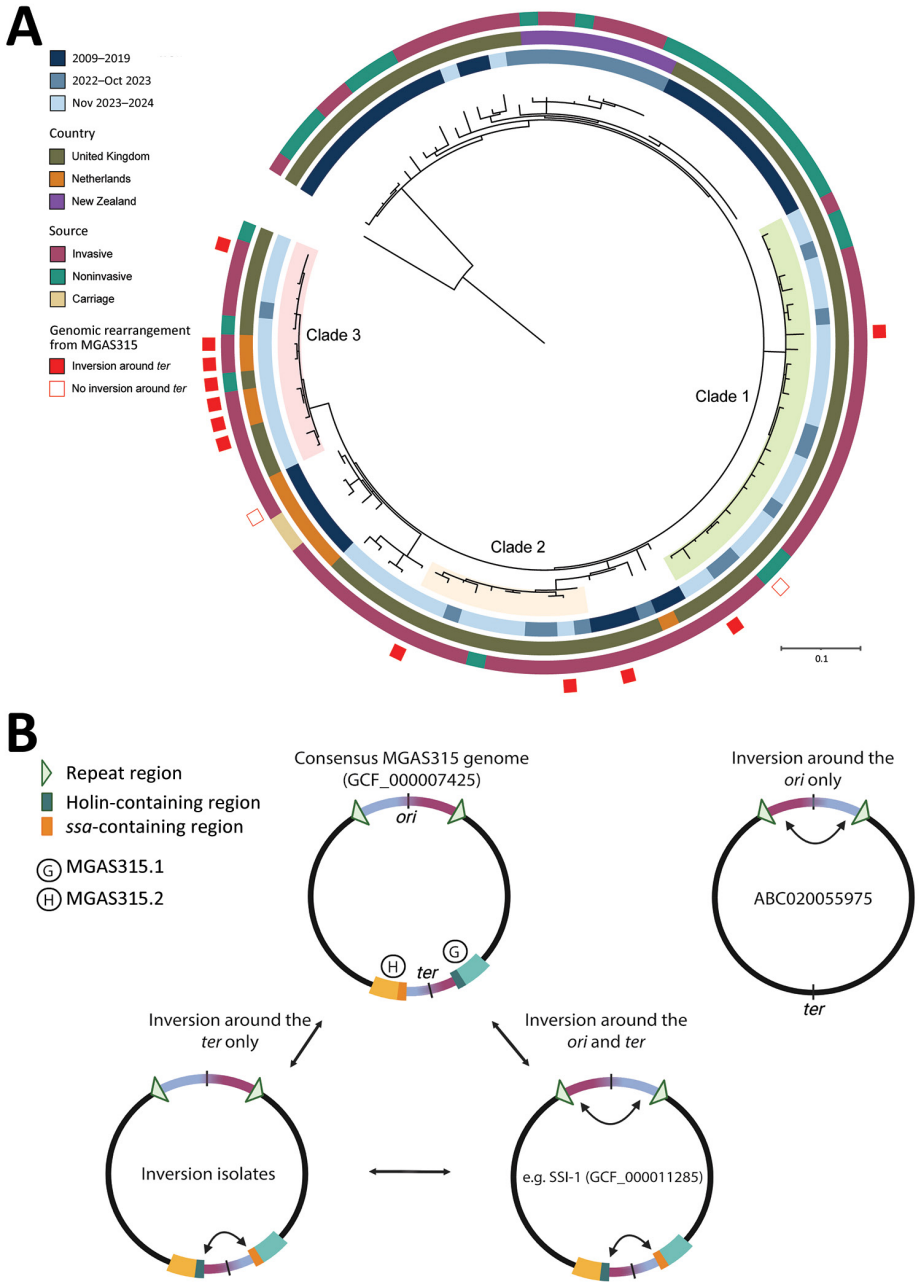
Genetic information about *emm*3.93 *Streptococcus pyogenes* isolates collected in the Netherlands and England, February 1, 2023–March 31, 2024, and reference isolates. A) Maximum-likelihood phylogenetic tree of 114 parsimony informative sites and 255 distinct site patterns from the core genome single-nucleotide polymorphism alignment of 104 *emm*3.93 isolates from the Netherlands, England, and New Zealand (2009–2024), compared with MGAS315 reference genome (RefSeq accession no. GCF_000007425). The tree was rooted on MGAS315, and colored rings outside the tree represent year of isolation, country, source, and presence of genomic inversion around the *ter*. Scale bar represents substitutions per site. B) Schematic of the 4 genomic conformations detected in the *emm*3 lineage. When compared with the consensus reference genome of strain MGAS315, an inversion around both the *ori* and *ter*, or around the *ter* alone (as detected in 11/12 surge isolates) is observed. Genome ABC020055975 indicates a strain lacking prophages MGAS315.1 and MGAS315.2 but with a detected inversion around the *ori*. G, MGAS315.1; H, MGAS315.2.

Phylogenetic analysis of the core SNP genomes of 104 *emm*3.93 of ST315 from the Netherlands, England, and New Zealand showed that recently-expanded *emm*3.93 isolates clustered into 3 distinct clades (Figure 3, panel A). Clades were determined as clusters containing <10 core SNPs difference whereas between-clade SNP distance was typically 20–50 SNPs (Appendix Figure 1). Lineage-defining SNPs were determined by clade-specific SNP annotations against reference strain MGAS315 (Appendix Table 2). Clades 1 and 2 only contained isolates from England, whereas clade 3 consisted of a mix of isolates from the Netherlands and England (Figure 3, panel A).

We explored possible genomic rearrangements because previous studies have described distinct genome configurations among *emm*3 consensus reference genomes MGAS315 and SSI-1, where SSI-1 contained large-scale genomic rearrangements compared with MGAS315 (*23*). Hybrid genome assembly for 14 *S. pyogenes emm*3.93 isolates (5 from the Netherlands and 9 from England) revealed that 12 isolates shared a genomic configuration that differed from MGAS315 by a ≈200-kb inversion around the terminus (*ter*) and from SSI-1 by a ≈521-kb inversion around the origin of replication (*ori*) (Figure 3, panel B; Appendix Figure 2). Of the 12 isolates, 11 belonged to *emm*3.93 surge isolates in each of the 3 clades and 1 was obtained from a patient with invasive disease from 2017 in England (Figure 3, panel A). The 200-kb genomic inversion around the *ter* is prophage-mediated and results in the movement of phage-encoded superantigen gene *ssa* and *holin* genes between prophages MGAS315.1 and MGAS315.2 (*24*) (Figure 3, panel B). The ≈521-kb inversion around the *ori* is spanned by 2 repeat regions of 6,200 bp (Figure 3, panel B). The inversions around the *ori* and the *ter* can occur independently, as indicated by strain ABC020055675, which lacks prophages MGAS315.1 and MGAS315.2 (Figure 3, panel B; Appendix Figure 2). Of interest, the genomic configuration of the remaining 2 isolates was identical to the consensus MGAS315 genome (Appendix Figure 2). One isolate was obtained from a patient with invasive disease in 2017 in the Netherlands with a common ancestor to clade 3 and the other from a patient with noninvasive infection from clade 1 (Figure 3, panel A). Although we analyzed a limited number of strains, our observations suggest the described genome configuration in the 11 recent *emm*3.93 isolates confers a survival advantage to *S. pyogenes*.

## Discussion

We report the rapid synchronous upsurge of *S. pyogenes emm* type 3.93 in the Netherlands and England during November 2023–March 2024. Epidemiologic analysis showed that *emm*3.93 was isolated more often from children 6–17 years of age with iGAS in both countries. The data from the Netherlands revealed a significant association between *emm*3.93 and pneumonia or pleural empyema, which was corroborated by an increase of *emm*3.93 among pleural isolates in England. Although the numbers were low, *emm*3.93 showed an increased risk for causing meningitis, which was significant in the data from England. Furthermore, *emm*3.93 was associated with streptococcal toxic shock syndrome in the Netherlands. Despite those concerning associations, the data from England showed no significant elevation in 7-day mortality for *emm*3.93 compared with mortality associated with other contemporaneous *emm* types.

Genomic analysis revealed 3 distinct clades among recent *emm*3.93 surge isolates. Clades 1 and 2 consisted only of isolates from England, whereas *emm*3.93 clade 3 contained a mix of isolates from the Netherlands and England. This analysis contrasts with previous analysis of the post–COVID-19 *emm*1 iGAS isolates, which showed predominantly country-specific surges of *emm*1 and little evidence of transmission of UK isolates to the Netherlands (*4*,*5*). This finding suggests that *emm*3.93 has had cross-border transmission events.

Analysis of complete genomes revealed a shared genomic configuration in all but 1 recent isolate from the 3 observed clades. When compared with other publicly available *emm*3 consensus reference genomes, the genomic configuration differed by either a ≈200-kb inversion around the *ter* or a ≈521-kb inversion around the *ori*. An identical genomic configuration was also identified in an invasive *emm*3.93 isolate from England from 2017, suggesting that this genome configuration was already circulating in the population before the surge. Of interest, *emm*3.93 caused a small surge of iGAS in England in 2018 (*22*) but did not expand to the levels observed in 2023–2024, nor did other countries report an increase in iGAS caused by *emm*3.93. We speculate that this 2017 isolate from England could have been a precursor to the 2023–2024 surge isolates but did not have a major advantage in the pre–COVID-19 climate because of sufficient population immunity.

Inverted repeats between prophages MGAS315.1 and MGAS315.2 have likely permitted the observed prophage-mediated homologous recombination. This phenomenon has been observed before in *emm*3.1 strains, which contained an alternative large-scale genomic rearrangement of 1 Mb because of chromosomal inverted repeats and was associated with invasive disease (*23*). However, because the inversion around the *ori* is also detected in an isolate without prophages MGAS315.1 and MGAS315.2 and without the inversion around the *ter*, we propose an updated view of the relationship between genomic configurations within *emm*3 *S. pyogenes*.

Because only single complete genomes are available of each of the reference strains, only a snapshot of the genomic configuration is being represented and then determined as consensus. Although our analysis of the individual isolates did not indicate a mixed population of inverted and noninverted genomes, care should be taken when interpreting the stability of an inversion that may occur in recombination hot spots with inverted homologous regions (*25*), because the switch back to its previous configuration may occur stochastically. For example, a recent emergent *emm*82 strain was detected to display an array of genome configurations among closely related isolates (*26*). Regardless, further information on the frequencies and selective pressures that cause inversions needs to be determined to make strong associations with epidemiologic data. Our dataset provided 2 observations that may suggest the observed genome configuration in the surge isolates conferred a survival benefit or affected virulence (*27*). First, an isolate from 2017 sharing a close common ancestor with the strains in clade 3 lacked the ≈200-kb inversion around the terminus and did not expand among iGAS patients. Second, we identified a single recent isolate in clade 1 without the surge-associated genomic configuration. Because the isolate was collected from a noninvasive wound swab, we speculate that specific genomic inversions are preferentially selected for during an infection pathway. The orientation in the recent surge isolates may affect virulence potential by shifting the location of the phage-encoded superantigen gene *ssa*, which was previously shown to be pyrogenic and toxic in rabbits (*28*). Additional research is needed to demonstrate if the observed genomic inversion affects *ssa* expression. Regardless, this clade 1 isolate demonstrates the dynamic nature and stochasticity of genome rearrangements within the *emm*3 population.

The first limitation of our study is the difference between the clinical data collected in the Netherlands and England with the lack of formal registration for death after infection in the Netherlands. The second limitation is the low number of genomes sequenced from isolates from the Netherlands collected during the recent outbreak. Although all 4 recent *emm*3.93 isolates from the Netherlands are in a single clade, other nonsequenced isolates might fall into the other outbreak-associated clades.

In conclusion, combined epidemiologic and molecular surveillance is key to detect emergence of new or more virulent *S. pyogenes* variants and to rapidly assess their clinical and epidemiologic relevance. *emm* typing revealed a replacement of *emm*1.0 by *emm*3.93 as the dominant type from November 2023 onwards, continuing over a few months. It is possible that population immunity to *emm*1.0 developed during 2023, leaving increased susceptibility to potentially new virulence traits of the new clades of *emm*3.93. Alternatively, the genomic inversion may have resulted in a competitive advantage of *emm*3.93 over *emm*1.0. In the context of the current upsurge of *S. pyogenes emm*3.93, international collaboration proved invaluable to assess the spread of the new clades and provide a more comprehensive picture of the associations of *emm*3.93 with both clinical manifestations and case fatality. In addition, a combination of long- and short-read sequencing was necessary to reveal the extent of genetic diversity for the emerged *emm*3.93 clades.

Appendix Additional information about *Streptococcus pyogenes emm* type 3.93 emergence, the Netherlands and England.
