# Supplementary material for: Streptococcus pyogenes emm Type 3.93 Emergence, the Netherlands and England
Source: Emerg Infect Dis. 2025 Feb;31(2):228–36. doi: 10.3201/eid3102.240880 (PMC11845126; doi:10.3201/eid3102.240880)
Supplement: Appendix — Additional information about Streptococcus pyogenes emm type 3.93 emergence, the Netherlands and England. [file 24-0880-Techapp-s1.pdf]

Article DOI: https://doi.org/10.3201/eid3102.240880

EID cannot ensure accessibility for supplementary materials supplied by authors. Readers who have difficulty accessing supplementary content should contact the authors for assistance.

Streptococcus pyogenes emm Type 3.93 Emergence, the Netherlands and England

Appendix

Appendix Table 1.

Sample ID	Country	Colonization type	*Emm* type	Collection year	Collection date period*	Source	BioProject	Sample accession
1138712	Netherlands	Invasive	3.93	2017	3	Blood	PRJNA1125189	SAMN41885169
4612210	Netherlands	Invasive	3.93	2017	3	Blood	PRJNA1125189	SAMN41885170
9877795	Netherlands	Invasive	3.93	2017	3	CSF	PRJNA1125189	SAMN41885171
4117681	Netherlands	Invasive	3.93	2019	4	Blood	PRJNA1125189	SAMN41885172
1221089	Netherlands	Invasive	3.93	2023	7	Blood	PRJNA1125189	SAMN41885173
6101976	Netherlands	Invasive	3.93	2024	7	CSF	PRJNA1125189	SAMN41885174
1157077	Netherlands	Invasive	3.93	2024	7	Sputum	PRJNA1125189	SAMN41885175
1111116	Netherlands	Invasive	3.93	2024	7	CSF	PRJNA1125189	SAMN41885176
1001106	Netherlands	Carriage	3.93	2017	3	Nose	PRJNA1125189	SAMN41885177
4610265	Netherlands	Carriage	3.93	2017	3	Nose	PRJNA1125189	SAMN41885178
1211118	Netherlands	Invasive	3.93	2016	3	Blood	PRJNA1125189	SAMN41885179
511605	England	Invasive	3.93	2017	3	Blood	PRJNA1125189	SAMN41885180
511606	England	Invasive	3.93	2017	3	Blood	PRJNA1125189	SAMN41885181
511608	England	Invasive	3.93	2018	4	Blood	PRJNA1125189	SAMN41885182
511614	England	Invasive	3.93	2018	4	Blood	PRJNA1125189	SAMN41885183
511619	England	Invasive	3.93	2018	4	Blood	PRJNA1125189	SAMN41885184
537090	England	Invasive	3.93	2018	4	Blood	PRJNA1125189	SAMN41885185
705173	England	Invasive	3.93	2018	4	Tissue	PRJNA1125189	SAMN41885186
1110171	England	Invasive	3.93	2023	6	Blood	PRJNA1125189	SAMN41885187
1112763	England	Invasive	3.93	2023	6	Blood	PRJNA1125189	SAMN41885188
1112829	England	Invasive	3.93	2023	6	Blood	PRJNA1125189	SAMN41885189
1154616	England	Invasive	3.93	2023	6	Tissue	PRJNA1125189	SAMN41885190
1154654	England	Invasive	3.93	2023	6	Blood	PRJNA1125189	SAMN41885191
1154693	England	Invasive	3.93	2023	6	Tissue	PRJNA1125189	SAMN41885192
1164292	England	Invasive	3.93	2023	6	Abscess	PRJNA1125189	SAMN41885193
1188675	England	Invasive	3.93	2023	6	Blood	PRJNA1125189	SAMN41885194
1193701	England	Invasive	3.93	2023	6	Pus	PRJNA1125189	SAMN41885195
1193716	England	Non-invasive	3.93	2023	6	Wound swab	PRJNA1125189	SAMN41885196
1200347	England	Invasive	3.93	2023	6	Blood	PRJNA1125189	SAMN41885197
1206953	England	Invasive	3.93	2023	6	Blood	PRJNA1125189	SAMN41885198
1219862	England	Invasive	3.93	2023	6	Blood	PRJNA1125189	SAMN41885199
1314957	England	Invasive	3.93	2023	7	Blood	PRJNA1125189	SAMN41885200
1329712	England	Invasive	3.93	2023	7	Blood	PRJNA1125189	SAMN41885201
1339736	England	Invasive	3.93	2023	7	Blood	PRJNA1125189	SAMN41885202
1339738	England	Invasive	3.93	2023	7	Blood	PRJNA1125189	SAMN41885203
1355993	England	Invasive	3.93	2023	7	Blood	PRJNA1125189	SAMN41885204
1355999	England	Non-invasive	3.93	2023	7	Throat swab	PRJNA1125189	SAMN41885205
1356031	England	Invasive	3.93	2023	7	Blood	PRJNA1125189	SAMN41885206
1377859	England	Invasive	3.93	2023	7	Blood	PRJNA1125189	SAMN41885207
1377860	England	Invasive	3.93	2023	7	Blood	PRJNA1125189	SAMN41885208
1380021	England	Invasive	3.93	2024	7	Blood	PRJNA1125189	SAMN41885209
1380060	England	Invasive	3.93	2024	7	Blood	PRJNA1125189	SAMN41885210
1380103	England	Invasive	3.93	2024	7	Blood	PRJNA1125189	SAMN41885211
1389677	England	Invasive	3.93	2024	7	Blood	PRJNA1125189	SAMN41885212
1389695	England	Invasive	3.93	2024	7	CSF	PRJNA1125189	SAMN41885213
1389698	England	Invasive	3.93	2024	7	Pleural fluid	PRJNA1125189	SAMN41885214

Sample ID	Country	Colonization type	*Emm* type	Collection year	Collection date period*	Source	BioProject	Sample accession
1389710	England	Invasive	3.93	2024	7	Blood	PRJNA1125189	SAMN41885215
1389712	England	Non-invasive	3.93	2024	7	Ear swab	PRJNA1125189	SAMN41885216
1389743	England	Invasive	3.93	2024	7	Tissue	PRJNA1125189	SAMN41885217
1397800	England	Non-invasive	3.93	2024	7	Vaginal swab	PRJNA1125189	SAMN41885218
1397823	England	Non-invasive	3.93	2024	7	Wound swab	PRJNA1125189	SAMN41885219
1397858	England	Invasive	3.93	2024	7	Blood	PRJNA1125189	SAMN41885220
1397882	England	Invasive	3.93	2024	7	Blood	PRJNA1125189	SAMN41885221
1413895	England	Non-invasive	3.93	2024	7	Wound swab	PRJNA1125189	SAMN41885222
1414070	England	Invasive	3.93	2024	7	Blood	PRJNA1125189	SAMN41885223
1417878	England	Invasive	3.93	2024	7	Blood	PRJNA1125189	SAMN41885224
1417895	England	Invasive	3.93	2024	7	Blood	PRJNA1125189	SAMN41885225
1420627	England	Invasive	3.93	2024	7	Blood	PRJNA1125189	SAMN41885226
1420644	England	Invasive	3.93	2024	7	Pleural fluid	PRJNA1125189	SAMN41885227
1420649	England	Non-invasive	3.93	2024	7	Skin swab	PRJNA1125189	SAMN41885228
1426265	England	Invasive	3.93	2024	7	Blood	PRJNA1125189	SAMN41885229
1426268	England	Invasive	3.93	2024	7	Blood	PRJNA1125189	SAMN41885230
1426286	England	Non-invasive	3.93	2024	7	Wound swab	PRJNA1125189	SAMN41885231
1426295	England	Invasive	3.93	2024	7	Blood	PRJNA1125189	SAMN41885232
1426298	England	Invasive	3.93	2024	7	Blood	PRJNA1125189	SAMN41885233
1426315	England	Invasive	3.93	2024	7	Blood	PRJNA1125189	SAMN41885234
1427257	England	Invasive	3.93	2024	7	Aspirate	PRJNA1125189	SAMN41885235
1433694	England	Invasive	3.93	2024	7	Blood	PRJNA1125189	SAMN41885236
1441513	England	Invasive	3.93	2024	7	Blood	PRJNA1125189	SAMN41885237
1441515	England	Invasive	3.93	2024	7	Blood	PRJNA1125189	SAMN41885238
1441543	England	Invasive	3.93	2024	7	Blood	PRJNA1125189	SAMN41885239
1441558	England	Invasive	3.93	2024	7	Blood	PRJNA1125189	SAMN41885240
1441578	England	Invasive	3.93	2024	7	Post mortem swab	PRJNA1125189	SAMN41885241
1822122	England	Invasive	3.93	2023	6	Blood	PRJNA1125189	SAMN41885242
1904308	England	Invasive	3.93	2018	4	Pleural fluid	PRJNA1125189	SAMN41885243
C_CC09_C_007	England	Non-invasive	3.93	2019	4	Cough plate	PRJEB43915	SAMEA8426085
C_CC09_H_010	England	Non-invasive	3.93	2019	4	Hand swab	PRJEB43915	SAMEA8426086
C_CC09_T_007	England	Non-invasive	3.93	2019	4	Throat swab	PRJEB43915	SAMEA8426087
C_CC38_T_010	England	Non-invasive	3.93	2019	4	Throat swab	PRJEB43915	SAMEA8426108
C_CC42_T_010	England	Non-invasive	3.93	2019	4	Throat swab	PRJEB43915	SAMEA8426116
C_E1_S_010	England	Non-invasive	3.93	2019	4	Settle plate	PRJEB43915	SAMEA8426124
C_E3_S_001	England	Non-invasive	3.93	2019	4	Settle plate	PRJEB43915	SAMEA8426125
C_E4_S_007	England	Non-invasive	3.93	2019	4	Settle plate	PRJEB43915	SAMEA8426126
C_E4_S_010	England	Non-invasive	3.93	2019	4	Settle plate	PRJEB43915	SAMEA8426127
C_H2_T_010	England	Non-invasive	3.93	2019	4	Throat swab	PRJEB43915	SAMEA8426128
ERR1359363	England	Non-invasive	3.93	2014	2	NA	PRJEB13551	SAMEA3930736
ERR1359409	England	Invasive	3.93	2014	2	NA	PRJEB13551	SAMEA3930992
ERR1359410	England	Invasive	3.93	2014	2	NA	PRJEB13551	SAMEA3931030
ERR1359485	England	Non-invasive	3.93	2014	2	NA	PRJEB13551	SAMEA3930732
ERR1359601	England	Non-invasive	3.93	2014	2	NA	PRJEB13551	SAMEA3930878

Sample ID	Country	Colonization type	*Emm* type	Collection year	Collection date period*	Source	BioProject	Sample accession
ERR1359650	England	Invasive	3.93	2014	2	NA	PRJEB13551	SAMEA3931105
ERR1359731	England	Non-invasive	3.93	2014	2	NA	PRJEB13551	SAMEA3930761
ERR1359759	England	Non-invasive	3.93	2014	2	NA	PRJEB13551	SAMEA3930584
ERR1359763	England	Non-invasive	3.93	2014	2	NA	PRJEB13551	SAMEA3930723
ERR1359862	England	Non-invasive	3.93	2014	2	NA	PRJEB13551	SAMEA3930781
23GA0015	New Zealand	Non-invasive	3.93	2023	6	Throat	PRJNA1100230	SAMN41032505
23GA0506	New Zealand	Invasive	3.93	2023	6	Blood	PRJNA1100230	SAMN41032978
23GA0008	New Zealand	Invasive	3.93	2023	6	Blood	PRJNA1100230	SAMN41032498
23GA0256	New Zealand	Non-invasive	3.93	2023	6	Throat	PRJNA1100230	SAMN41032739
23GA0600	New Zealand	Invasive	3.93	2023	6	Blood	PRJNA1100230	SAMN41033069
23GA0688	New Zealand	Invasive	3.93	2023	6	Blood	PRJNA1100230	SAMN41033151
23GA0435	New Zealand	Invasive	3.93	2023	6	Blood	PRJNA1100230	SAMN41032912
23GA0458	New Zealand	Invasive	3.93	2023	6	Blood	PRJNA1100230	SAMN41032934
23GA0432	New Zealand	Non-invasive	3.93	2023	6	Tissue	PRJNA1100230	SAMN41032909

*1 = 2009–2011; 2 = 2012–2014; 3 = 2015–2017; 4 = 2018–2019; 5 = 2020–2021; 6 = 2022-Oct 2023; 7 = Nov 2023–2024

Appendix Table 2. Lineage-specific single nucleotide polymorphisms (SNPs). Clade-specific SNP annotations against reference strain (Ref) MGAS315 (NC_004070.1), determined with Snippy.

Clade	MGAS315 Location	Locus tag	Gene	Product	S/NS	Amino acid change	Ref	SNP/indel
Clade 1 (26 isolates)	298891	SPYM3_RS01565	yidC	membrane protein insertase YidC	NS	Arg305Gln	C	T
	347532	Intergenic	NA	NA	Intergenic	NA	CA	C
	401009	SPYM3_RS02125	ccpA	catabolite control protein A	NS	Ala167Asp	C	A
	433606	SPYM3_RS10295	NA	DUF2326 domain-containing protein	Frameshift	Lys158fs	AT	A
	529296	Intergenic	NA	NA	Intergenic	NA	A	G
	591410	Intergenic	NA	NA	Intergenic	NA	AG	A
	700812	SPYM3_RS03535	recJ	single-stranded-DNA-specific exonuclease RecJ	NS	Met315Val	A	G
	799621	SPYM3_RS04090	NA	response regulator transcription factor	NS	Gly82Ser	G	A
	810674	SPYM3_RS04135	pbp3	D-alanyl-D-alanine carboxypeptidase PBP3	NS	Thr265Ser	C	G
	947767	SPYM3_RS04805	NA	restriction endonuclease subunit S	S	Gly48Gly	G	C
	1068134	SPYM3_RS05460	NA	methionyl aminopeptidase	S	Leu255Leu	T	C
	1217111	Intergenic	NA	NA	Intergenic	NA	T	C
	1434583	SPYM3_RS07490	NA	PBSX family phage terminase large subunit	NS	Phe266Tyr	A	T
	1581156	SPYM3_RS08200	NA	magnesium transporter CorA family protein	S	Leu262Leu	A	G
	1609622	SPYM3_RS08345	NA	MerR family transcriptional regulator	S	Asp116Asp	G	A
Clade 2 (11 isolates)	184143	SPYM3_RS01030	speG	streptococcal pyrogenic exotoxin SpeG	S	Ser205Ser	T	C
	642839	SPYM3_RS03245	ideS	immunoglobulin G-degrading enzyme IdeS	NS	Glu66Lys	C	T
	833981	Intergenic	NA	NA	Intergenic	NA	T	TA
	1147260	SPYM3_RS05820		hypothetical protein	NS	Thr59Ile	G	A
	1524461	SPYM3_RS07970	grpE	nucleotide exchange factor GrpE	NS	Leu103Ile	G	T
	1740613†	SPYM3_RS08960	NA	pneumococcal-type histidine triad protein	NS	Ala172Val	G	A
	1763604	SPYM3_RS09050	ropB	quorum-sensing system transcriptional regulator RopB/Rgg1	NS	Asp24His	G	C
Clade 3 (14 isolates)	341961	SPYM3_RS01800	metG	methionine–tRNA ligase	S	Pro229Pro	A	C
	370021‡	SPYM3_RS01945	msrA	peptide-methionine (S)-S-oxide reductase MsrA	NS	Ala158Thr	G	A

Clade	MGAS315 Location	Locus tag	Gene	Product	S/NS	Amino acid change	Ref	SNP/indel
	427400	SPYM3_RS02235	NA	serine kinase	NS	Asp145Gly	A	G
	502485	SPYM3_RS02620	NA	(S)-acetoin forming diacetyl reductase	S	Tyr150Tyr	C	T
	629815	SPYM3_RS03180	NA	cation diffusion facilitator family transporter	NS	Pro200Ser	G	A
	987708	SPYM3_RS05000	NA	phage tail spike protein	S	Arg79Arg	C	T
	987714	SPYM3_RS05000	NA	phage tail spike protein	S	delAGTGinsGGTA	CACT	TACC
	1270371	SPYM3_RS06610	NA	hypothetical protein	S	Asp117Asp	C	T
	1281283	SPYM3_RS06655	NA	shikimate kinase	NS	Asn171Lys	G	C
	1300051	SPYM3_RS06745	NA	ROK family protein	S	Asn105Asn	T	C
	1802116	SPYM3_RS09230	NA	APC family permease	NS	Thr122Ile	C	T

*Also present in the 2 most related isolates to the clade
†Missing from 1 isolate in the clade
‡Missing from 2 isolates in the clade
S refers to synonymous and NS refers to non-synonymous mutations

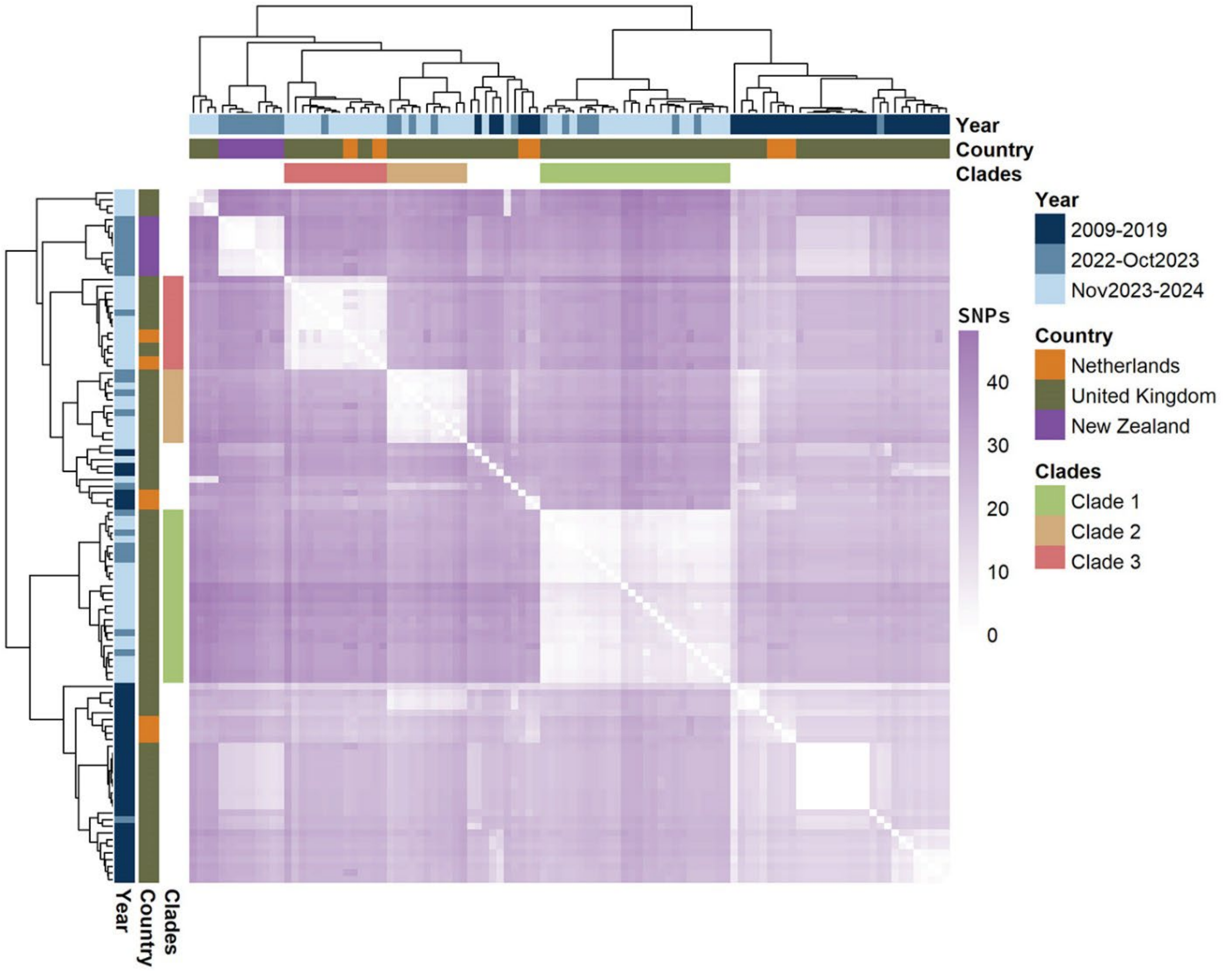

Appendix Figure 1. Core-SNP distance matrix of the core SNP alignment of 104 *emm3.93* genomes from the Netherlands, United Kingdom and New Zealand between 2009–2024. Color in the heatmap indicates the number of SNPs between isolates, with highly similar isolates in white and shifting toward purple as number of SNPs increases. Year, country, and *emm3.93* clades are visualized by both dendrograms, which have been clustered with default hierarchical clustering from R package pheatmap.

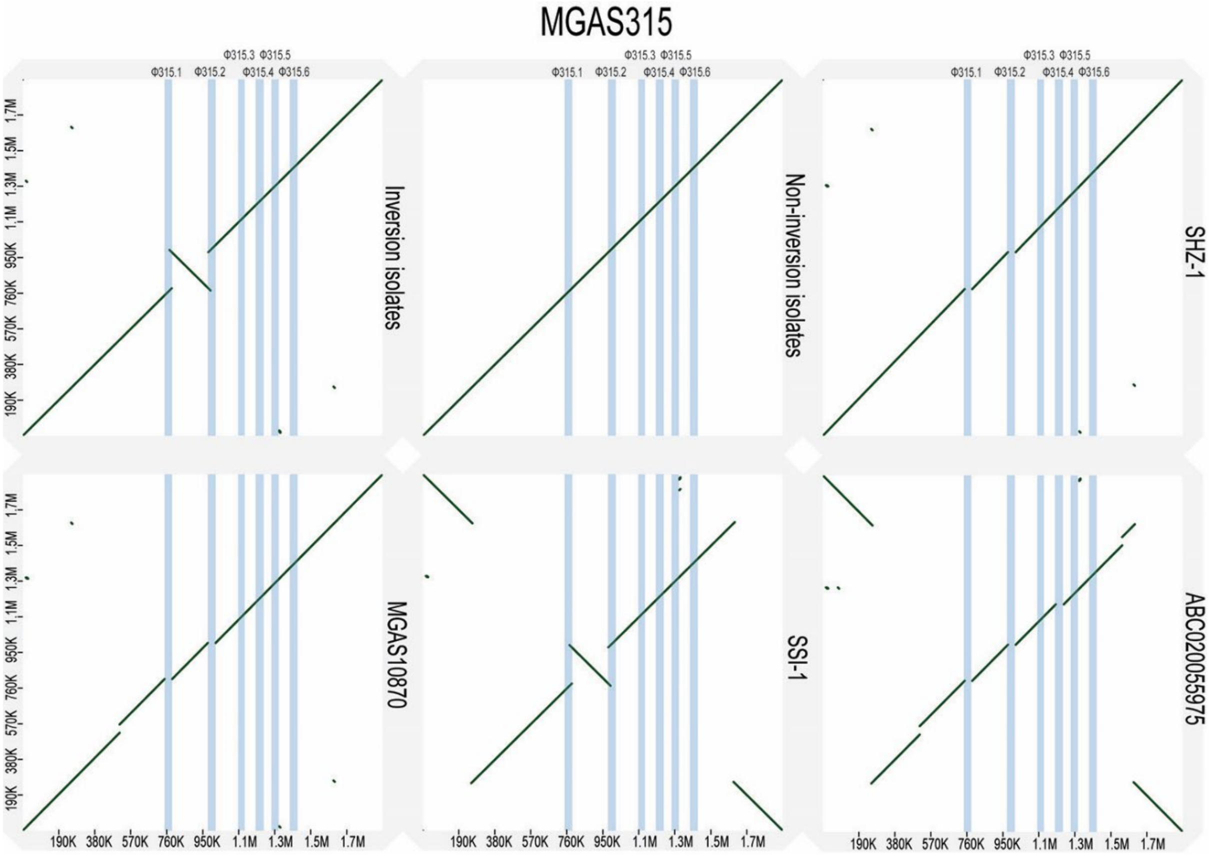

Appendix Figure 2. Dotplots representing pairwise genome alignment of MGAS315 reference genome (NC_004070; x-axes) against either inversion isolates, non-inversion isolates or four publicly available *emm3.1* complete genomes (y-axes). An upwards diagonal black line represents region of shared sequence in the same orientation, whereas a downwards diagonal black line represents region of shared sequence in the opposition orientation. Six known MGAS315 prophages (Φ315.1, Φ315.2, Φ315.3, Φ315.4, Φ315.5, Φ315.6) are visualized as blue vertical lines. White gaps represent no sequence similar shared between the genomes. SSI-1 genomic configuration is similar to NIH34, M3-b and STAB902 and therefore not displayed. Plots were generated by D-GENIES.
